# Biomechanical analysis of a novel height-adjustable nano-hydroxyapatite/polyamide-66 vertebral body: a finite element study

**DOI:** 10.1186/s13018-019-1432-2

**Published:** 2019-11-14

**Authors:** Guanghui Chen, Baoquan Xin, Mengchen Yin, Tianqi Fan, Jing Wang, Ting Wang, Guangjian Bai, Jianru Xiao, Tielong Liu

**Affiliations:** 1 Department of Orthopedics, Peking University Third Hospital, Beijing, China; 2 Department of Orthopaedic Oncology Center, Changzheng Hospital, Second Military Medical University, #415 Fengyang Road, Shanghai, 200003 China; 3 Department of Orthopedics, Longhua Hospital, 725 South Wanping Road, Shanghai, 200003 China

**Keywords:** Biomechanics, Finite element analysis, Corpectomy, Titanium mesh cage, Artificial vertebral body, Nano-hydroxyapatite/polyamide-66

## Abstract

**Background:**

To compare the biomechanical properties of a novel height-adjustable nano-hydroxyapatite/polyamide-66 vertebral body (HAVB) with the titanium mesh cage (TMC) and artificial vertebral body (AVB), and evaluate its biomechanical efficacy in spinal stability reconstruction.

**Methods:**

A 3D nonliner FE model of the intact L1-sacrum was established and validated. Three FE models which instrumented HAVB, TMC, and AVB were constructed for surgical simulation. A pure moment of 7.5 Nm and a 400-N preload were applied to the three FE models in 3D motion. The peak von Mises stress upon each prosthesis and the interfaced endplate was recorded for analysis. In addition, the overall and intersegmental range of motion (ROM) of each model was investigated to assess the efficacy of each model in spinal stability reconstruction.

**Results:**

AVB had the greatest stress concentration compared with TMC and HAVB in all motions (25.6–101.8 times of HAVB, 0.8–8.1 times of TMC). The peak stress on HAVB was 3.1–10.3% of TMC and 1.6–3.9% of AVB. The maximum stress values on L2 caudal and L4 cranial endplates are different between the three FE models: 0.9–1.9, 1.3–12.1, and 31.3–117.9 times of the intact model on L2 caudal endplates and 0.9–3.5, 7.2–31.5, and 10.3–56.4 times of the intact model on L4 cranial endplates in HAVB, TMC, and AVB, respectively, while the overall and segmental ROM reduction was similar between the three models, with AVB providing a relatively higher ROM reduction in all loading conditions (88.1–84.7% of intact model for overall ROM and 69.5–82.1% for L1/2, 87.0–91.7% for L2/4, and 71.1–87.2% for L4/5, respectively).

**Conclusions:**

HAVB had similar biomechanical efficacy in spinal stability reconstruction as compared with TMC and AVB. The material used and the anatomic design of HAVB can help avoid stress concentration and the stress shielding effect, thus greatly reducing the implant-associated complications. HAVB exhibited some advantageous biomechanical properties over TMC and AVB and may prove to be a potentially viable option for spinal stability reconstruction. Further in vivo and vitro studies are still required to validate our findings and conclusions.

## Background

Corpectomy is generally accepted as an effective surgical procedure for spinal cancer metastasis, infection, deformity, and traumatic injuries [1, 2]. However, restoration of the spinal column during surgery remains a technical challenge in clinical practice [3, 4]. To create a biological environment for fusion, rigid stabilization with an ideal vertebral body graft is highly recommended, and a number of interbody graft types have been constructed including allografts, cement, metal, and synthetic materials [5–9]. Among them, titanium mesh cages (TMC) and artificial vertebral body (AVB) such as the VLIFT cage made of metal alloy material have been used widely for their good mechanical properties [10–13]. However, an increasing number of studies have demonstrated that these types of implants are often associated with some troublesome implant-associated complications such as stress shielding, high subsidence rate, and fatigue failure [1, 14, 15].

Although some in vivo and in vitro studies have compared the biomechanical properties of the TMC and AVB systems and found no significant difference between them [16, 17], few studies have addressed the mechanisms underlying these implant-associated complications, and there is little knowledge about the stress acting inside these prostheses. In addition, the complications related to these two prostheses are not all the same; for instance, AVB has a higher subsidence and revision surgery rate than TMC, suggesting that there may exist different mechanical mechanisms in these two prostheses [1]. To the best of our knowledge, no study has reported the use of FEM analysis to investigate the biomechanical properties of these two prostheses.

Various types of bioceramics have been used for treating bone defects [7, 18–20], but they have some drawbacks such as weak mechanical properties and chemical stability, which limit their clinical applications [21]. To address these issues, many composite systems have been explored as bone substitute materials, including HA reinforced polyethylene, polylactide, collagen, and Polyactive™ [22–25]. To enhance HA bioceramic toughness, Wei and Li [26] employed a novel method to make biomaterial n-HA/PA66 composite and found that the composite with 64.25 wt% n-HA had excellent mechanical properties close to the natural bone.

Nano-hydroxyapatite/polyamide66 (n-HA/PA66) as a biomimetic biomaterial has been approved for clinical application for more than 10 years, and many products made of this material have been used for spinal reconstruction [26–28]. Additionally, some previous investigations have proved that n-HA/PA66 has a good clinical application with good mechanical performance in bony fusion [28, 29]. To reduce the incidence of implant-related complications, we have developed a novel height-adjustable vertebral body (HAVB) made of n-HA/PA66, which was reported in our previous study [30]. However, its biomechanical properties were not fully discussed in that paper because of limited data at that time. The objective of this study was to use FEM analysis to compare the biomechanical properties of this novel prosthesis with TMC and AVB, and evaluate their biomechanical efficacy in spinal stability reconstruction.

## Materials and methods

### FE model of the intact L1-sacrum spine

**Fig. 1:**
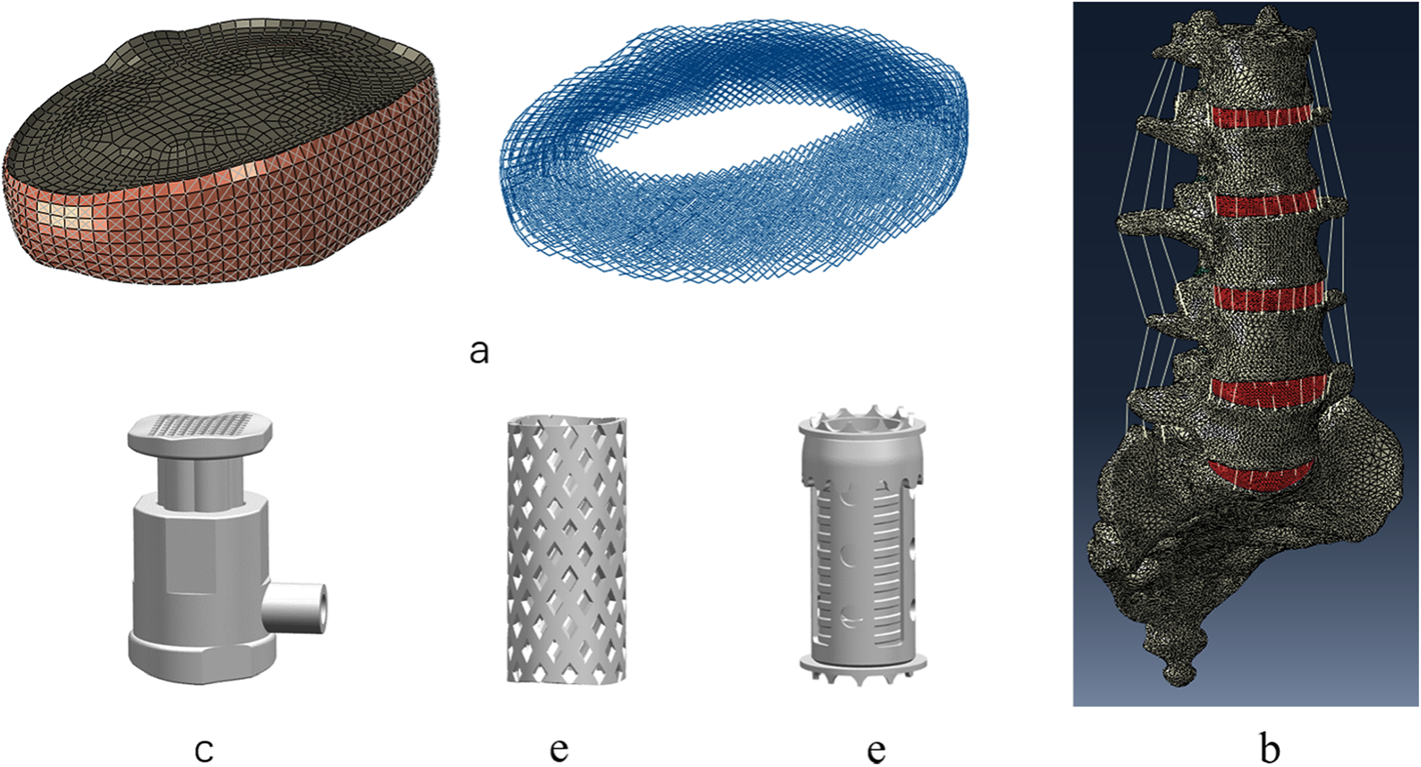
Construction of the nonliner 3D FE models and geometry of the three prostheses. **a** 3D FE model of the disc. **b** L1-sacrum vertebra. **c** Novel height-adjustable nano-hydroxyapatite/polyamide-66 vertebral body. **d** Titanium mesh cage. **e** Artificial vertebral body

To construct the model geometry, a high-resolution CT scan was obtained at 1-mm intervals in a 36-year-old healthy male. The obtained CT images severed as a DICOM format and were imported into the software Mimics 16.0 (Materialise Inc., Leuven, Belgium) to construct a three-dimensional (3D) model of the L1-sacrum. Software Hypermesh 13.0 (Altair Technologies, Inc., Fremont, CA, USA) was used to perform mesh generation, and Abaqus software (Abaqus 6.13, Karlsson & Sorenson, Inc., Providence, RI) was used for FEM simulation. The FEM consists of the cortical bone, cancellous bone, endplates, intervertebral discs, articular cartilage, and seven ligamentous systems including the anterior longitudinal ligament (ALL), posterior longitudinal ligament (PLL), ligamentous flavum (LF), capsular ligaments (CL), intertransverse ligaments (ITL), interspinous ligaments (ISL), and supraspinous ligaments (SSL) (Fig. 1a, b).

### Material properties

The material properties of the osseous tissues were assumed to be linear, isotropic, and homogeneous, and data were obtained from the literature. All the ligaments were modeled with truss elements and subjected to tensile load only. The cross-sectional area of each ligament was obtained from previous finite element studies [31]. The intervertebral discs were composed of 44% nucleus pulposus (NP) and 56% annulus fibrous (AF) tissue and reinforced by collagen fibers. Eight layers of collagen fibers were generated radially with a 30–45° angle from the horizontal surface and varied from the inner to outer lamina of the AF tissue. The friction coefficient of the facet joint was set at 0.1. The types of elements and material properties of each component are shown in Table 1.

**Table 1:** Material properties and element type used in the finite element models

Component	Element type	Young’s modulus (MPa)	Poisson’s ratio	Cross-sectional area (mm^2^)
Cortical bone	Tetrahedral	12,000	0.3	–
Cancellous bone	Tetrahedral	100	0.2	–
Pedicle	Tetrahedral	3500	0.25	–
Facet joints	Hexahedral	15	0.45	–
Endplate	Hexahedral	24	0.25	–
NP	Hexahedral	1	0.49	–
AF	Hexahedral	4.2	0.45	–
ALL	Truss	7.8	/	63.7
PLL	Truss	1	/	20
LF	Truss	1.5	/	40
CL	Truss	7.5	/	30
ITL	Truss	10	/	1.8
ISL	Truss	1	/	40
SSL	Truss	3	/	30
TMC	Tetrahedral	110,000	0.3	
AVB	Tetrahedral	110,000	0.3	
HAVB	Tetrahedral	4000	0.3	

*NP* nucleus pulposus, *AF* annulus fibrosus, *ALL* anterior longitudinal ligament, *PLL* posterior longitudinal ligament, *LF* ligamentum flavum, *CL* capsular ligament, *ITL* intertransverse ligament, *ISL* interspinous ligament, *SSL* supraspinous ligament, *TMC* titanium mesh cage, *AVB* artificial vertebral body, *HAVB* height-adjustable vertebral body

### FEM validation

Validation of the intact FEM was performed according to the protocol used in the cadaveric biomechanical study by Shim et al. [32] and Renner et al. to compare the range of motion (ROM) in flexion, extension, lateral bending, and axial rotation.

### Surgical FE models

**Fig. 2:**
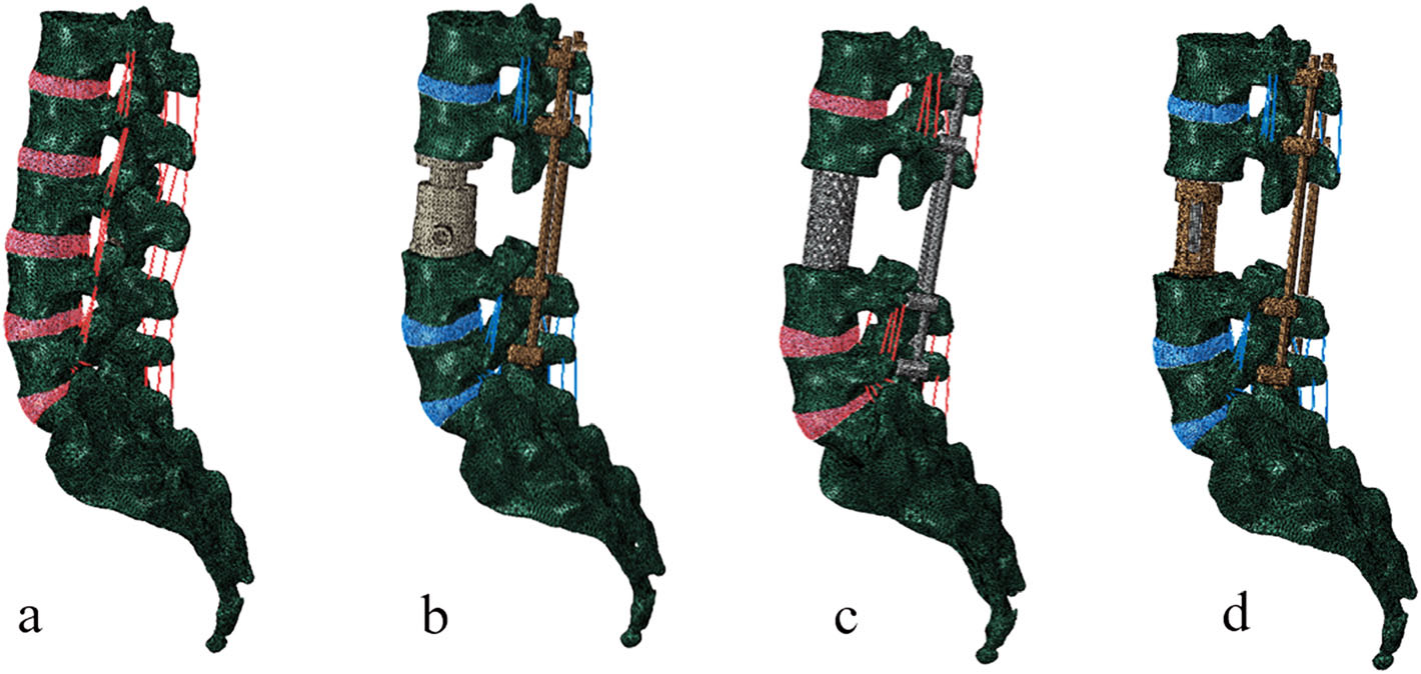
Simulation of the surgical procedure of L3 vertebral replacement. **a** The intact model. **b** HAVB with posterior pedicle screw and rod fixation. **c** TMC with posterior pedicle screw and rod fixation. **d** AVB with posterior pedicle screw and rod fixation

To simulate the surgical procedure, L3 corpectomy and vertebral body replacement combined with posterior fixation were performed. In the surgical segment, all L3 ligaments, and upper and lower endplates together with the L3 vertebra were removed. L1, L2, L4, and L5 were fixated with the pedicle screw rod system. 3D geometrical models of these three prostheses were created using commercial software (UG nx8.0, Siemens PLM Software, Germany) (Fig. 1c–e). The material of the HAVB was n-HA/PA66, and the other two implants were made of titanium alloy. The configuration of the HAVB was consistent with the description of our earlier study [17]. The intact FE model and three surgical FE models were constructed as shown in Fig. 2. The contact between the pedicle screw and the bone was set as an “embedded” coupling constraint, and prosthesis-endplate interface was modeled by surface-to-surface contact elements to simulate the early postoperative stage after spinal instrumentation. The friction coefficient at the prosthesis-endplate interface was 0.8 to mimic the rough contact interface.

### Boundary and loading conditions

FEM simulations were performed on the L1-sacrum. The sacrum of all these FE models was defined to be rigidly fixed, and the load was applied on the upper endplate of the L1 vertebral body. An axial preload of 400 N was used to mimic upper body weight, and a 7.5-Nm pure moment was applied to simulate flexion, extension, left/right lateral bending, and left/right rotation.

After numerical calculation, the overall and intersegmental ROM was recorded, and maximum von Mises stress on the implants and endplates was investigated and compared for analysis.

## Results

### Validation of the intact FEM

The ROM data of L2–3, L3–4, and L4–5 were obtained and compared with the results of Shim et al. from a cadaveric biomechanical study (Table 2). This intact FEM was confirmed to be valid, and the calculated ROM at each intervertebral segment was within ± 1° of the ROM values presented in Shim et al.’s [32] study in all motions (Additional file 1: Figure S1).

**Table 2:** Comparison of ROM at each intervertebral level between the current study and Shim et al.’s study

Intervertebral level	ROM (degrees)											
	Flexion		Extension		Left bending		Right bending		Left rotation		Right rotation	
	Current	Shim	Current	Shim	Current	Shim	Current	Shim	Current	Shim	Current	Shim
L2–3	5.494	4.7 (1)	3.946	3.6 (0.5)	3.202	3.3 (0.3)	3.374	3.3 (0.3)	4.532	4.1 (1.1)	4.394	4.1 (1.1)
L3–4	3.592	4.2 (0.8)	2.492	2.9 (0.5)	4.0337	3.5 (1)	4.2973	3.5 (1)	2.796	2.8 (0.6)	2.7101	2.8 (0.6)
L4–5	5.907	5.4 (0.9)	3.134	3.8 (1)	3.804	4.4 (1.1)	3.437	4.4 (1.1)	3.712	3.8 (1)	3.930	3.8 (1)

*ROM* range of motion

The number in the parentheses represents the standard errors

### Endplate and implant stress of the FEM

**Fig. 3:**
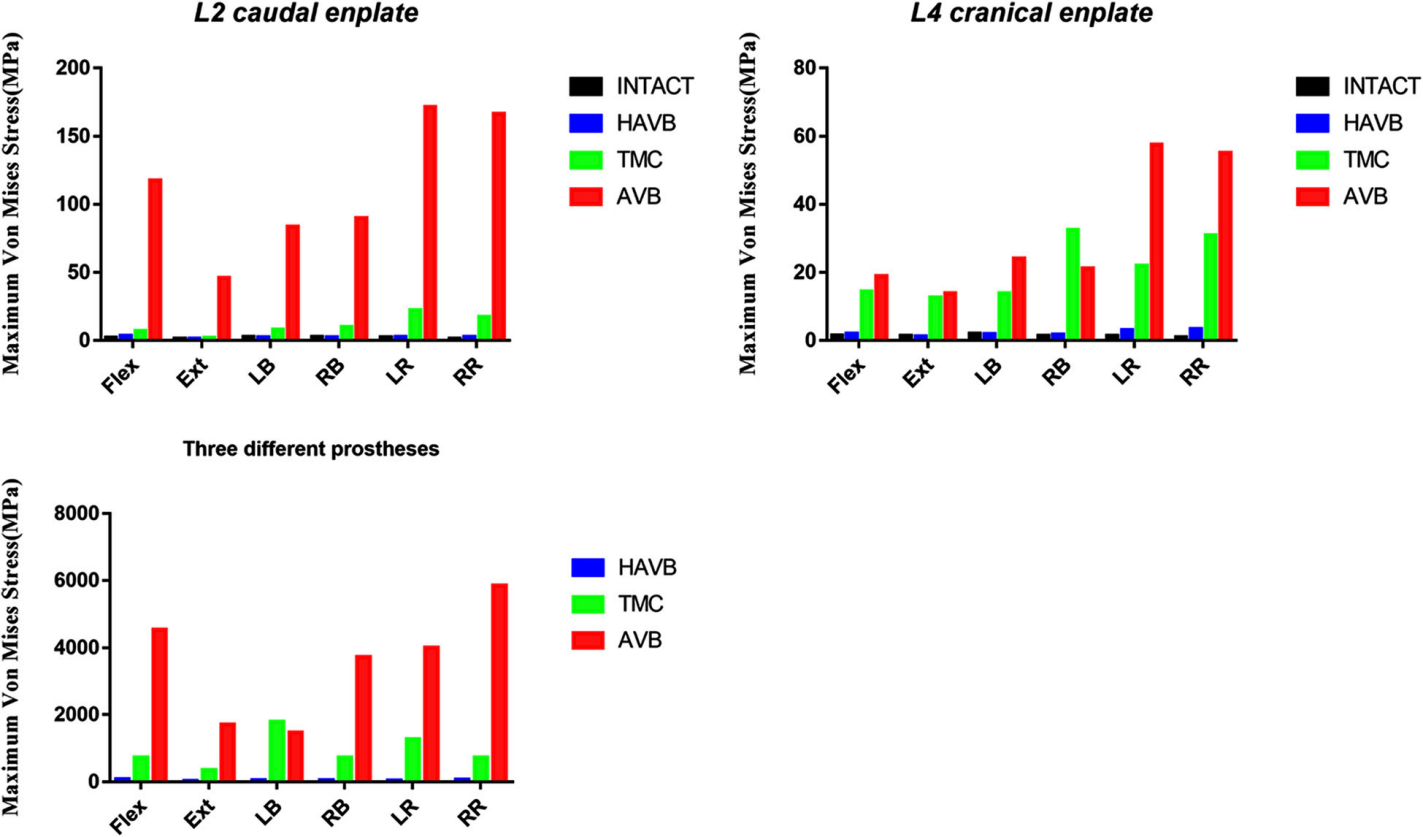
Comparison of maximum von Mises stress on L2 caudal endplate, L4 cranial endplate, and three different prostheses in six different working conditions. INTACT, the intact model; HAVB, height-adjustable vertebral body; TMC, titanium mesh cage; AVB, artificial vertebral body; Flex, flexion; Ext, extension; LB, left bending; RB, right bending; LR, left rotation; RR, right rotation

The maximum von Mises stress on the L2 caudal endplate and L4 cranial endplate was compared between the intact and surgical models (Fig. 3). In the HAVB model, the stress of the L2 caudal endplate increased by 63.2% in flexion, 9.8% in left rotation, and 8.5% in right rotation, and decreased by 7.2% in extension, 8.1% in left bending, and 4.9% in right bending. Similar stress change was applied to the L4 cranial endplate in the HAVB model. Either in TMC or in AVB model, the peak stress on the L2 caudal endplate and L4 cranial endplate was much higher in all motions than that in the HAVB model. In addition, compared with the TMC model, the stress of L2 caudal endplate in the AVB model was 15 times higher than that of TMC during flexion, 22.5 times higher than that of during extension, and 7–9 times higher than that of during other motions. As for the stress on the L4 cranial endplate, no such big difference was observed between them. Except for the apparently high stress in the left and right rotation in AVB vs. TMC, the value of stress change was almost on the same level in the other motions.

**Fig. 4:**
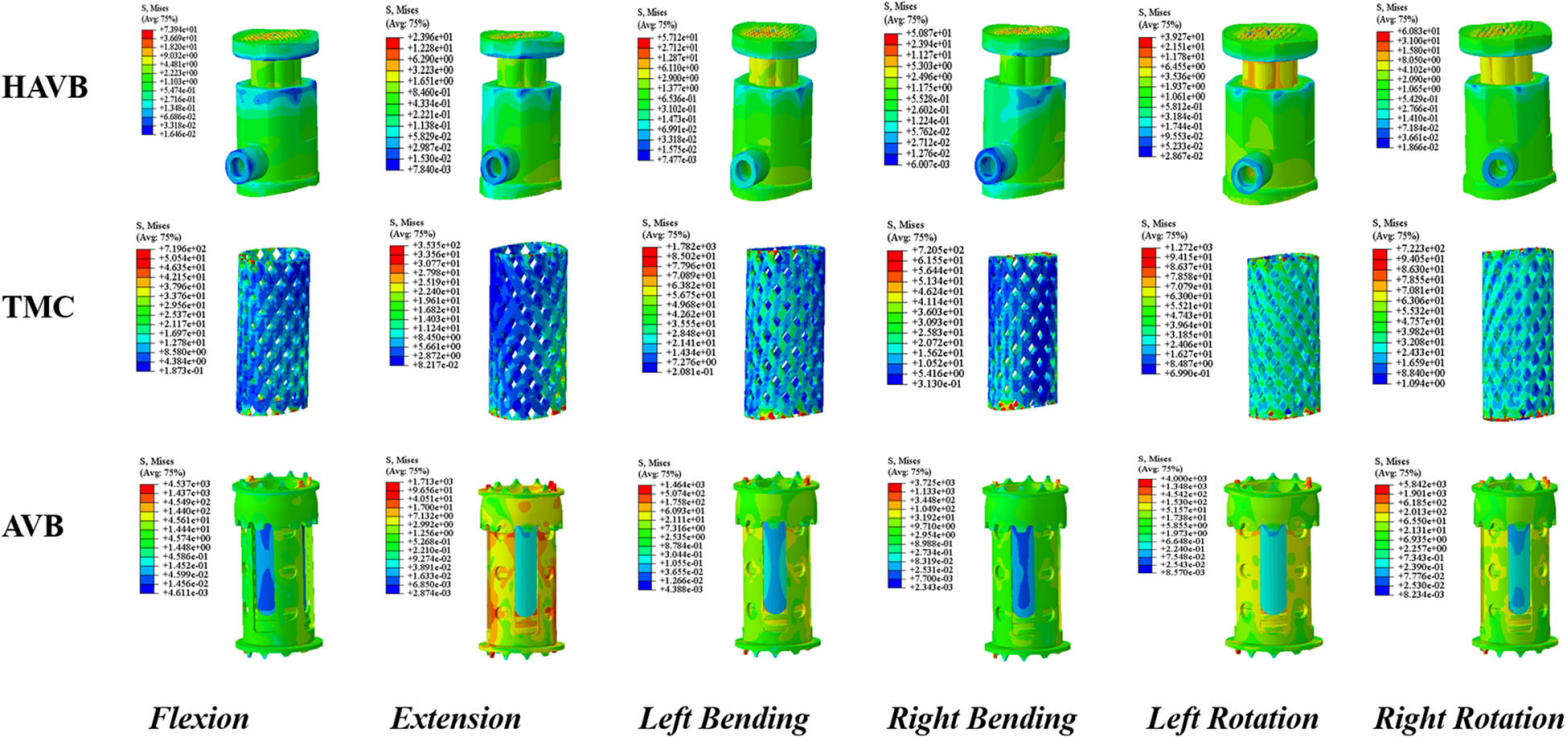
Stress distribution of HAVB, TMC, and AVB in flexion, extension, left bending, right bending, left rotation, and right rotation conditions

In addition, the peak stress on the TMC and AVB implants was much higher than that on the HAVB. A tremendous stress concentration was observed at the spike site of the TMC and AVB models. Stress distribution in the AVB model was significantly higher than that in the TMC model (Fig. 4).

### Range of motion

**Fig. 5:**
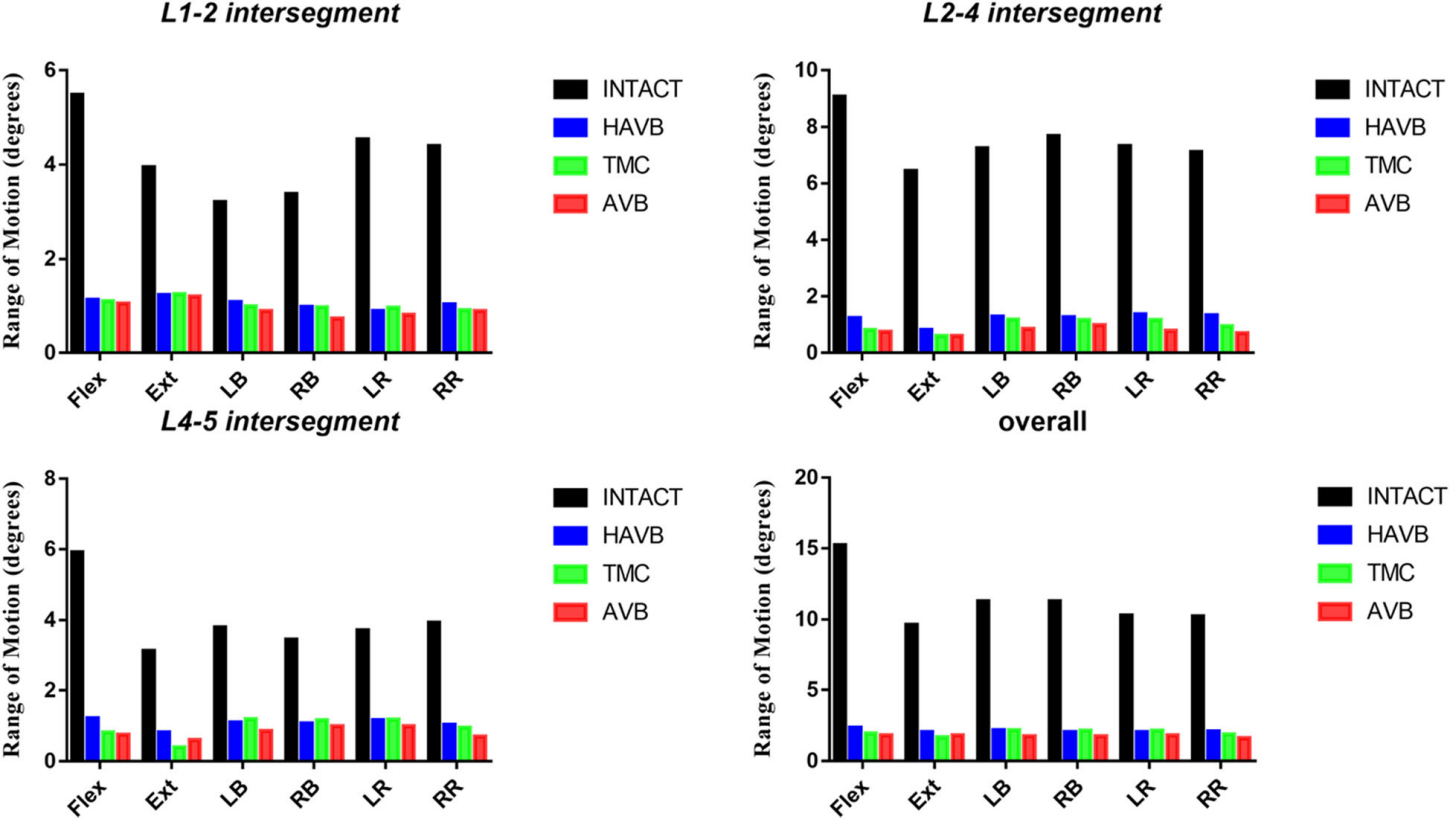
Comparison of overall and intersegmental ROM of four different FE models in six different working conditions. ROM, range of motion; INTACT, the intact model; HAVB, height-adjustable vertebral body; TT, titanium mesh; AVB, artificial vertebral body; Flex, flexion; Ext, extension; LB, left bending; RB, right bending; LR, left rotation; RR, right rotation

ROM at the surgical segments (L1/2, L2/4, and L4/5) was compared in all models (Fig. 5). Among them, the intact model showed the greatest ROM in all motions, while ROM reduced at L1/2 by 80% in flexion and rotation, 69% in extension, and 70% in left and right bending in the surgical models. No significant difference in ROM reduction was observed at this segment between these surgical models. At L4/5, flexion ROM reduced by 80%, 86%, and 87%, and extension ROM reduced by 74%, 87%, and 80% in HAVB, TMC, and AVB, respectively. In the other directions of motion, HAVB and TMC demonstrated an approximate 70% reduction, which was slightly lower than that of AVB. A greater ROM reduction was observed at L2/4 than that at L1/2 and L4/5 in all motions. Flexion ROM reduced by more than 91% in TMC and AVB vs. 86% in HAVB. In extension, TMC and AVB showed a similar ROM change in extension, with a reduction about 90%, which was higher than 87% of HAVB. In lateral bending, the ROM reduction in HAVB and TMC was 83%, and 88% in HAVB. In rotation, the ROM reduction was 81% in HAVB, 85% in TMC, and 90% in AVB. The overall flexion ROM reduction at L1–5 was 84%, 87%, and 88% in HAVB, TMC, and AVB, respectively. A similar trend of ROM change was observed in the other motions. Overall, more ROM restriction was observed in the AVB model in all motions.

## Discussion

Corpectomy has become a common surgical procedure for spinal tumors, deformity, infection, and trauma [1, 2]. Accordingly, various types of interbody cages have been developed and applied clinically. Among them, TMC and AVB have become the most commonly used prostheses due to their good biomechanical properties and a high fusion rate. However, with the increased number of patients and prolonged follow-up periods, more implant-associated complications have been reported [33]. To overcome the disadvantages of these spinal interbody prostheses, we have developed a novel HAVB prosthesis made of n-HA/PA66. The details of the design process were reported in our previous study [30]. However, we did not fully discuss its biomechanical efficacy in that paper because of the lack of sufficient evidence. In this study, we used the FE method to investigate the performance of HAVB in spinal stability reconstruction and compared its biomechanical properties with those of the TMC and AVB systems.

As TMC and AVB have been widely used in clinical practice, many in vitro cadaveric studies have been performed to verify the efficacy of TMC and AVB in spinal stability reconstruction [13]. To compare the in vitro biomechanical properties of three different expandable cages with a nonexpendable cage, Rober et al. [13] conducted a cadaveric study and reported that no significant difference could be determined. In contrast, Knop et al. [21] reported that Synex was associated with significantly higher stiffness and lower ROM for rotation and bending as compared with TMC. It was found in our study that AVB was associated with the greatest ROM reduction in all loading conditions but no significant difference in ROM reduction was observed as compared with HAVB and TMC, indicating that spinal stability of these three FE models is similar.

Cappuccino et al. [34] reported that additional bilateral pedicle screw and rod fixation could provide the maximum ROM reduction for single-level lumbar fusion. Many other studies have also emphasized the importance of additional posterior stabilization [17, 35]. In our study, a noticeable ROM reduction was observed in all instrumented FE models. The reduction of flexion ROM was relatively higher than that of extension ROM in all models, with ROM reduction being the greatest at L2–4 (86% for HAVB and 91% for TMC and AVB). Additionally, we observed that the stress distribution of flexion on the prosthesis was higher than that of extension, indicating that both the posterior fixation system and the anterior prosthesis played an important role in ROM reduction. As the spikes at both ends of the TMC and AVB systems are embedded into the endplate, it greatly restricts ROM of extension. Otherwise, AVB exhibited greater ROM rotation reduction than TMC and HAVB in overall model and all intersegments except in L1–2, indicating that the type of prosthesis plays a critical role in ROM reduction, which is consistent with the finding of Knop et al. [36]. All these results illustrate that both the prosthesis and posterior stabilization play an essential role in spinal stability and HAVB has the similar biomechanical efficacy in spinal stability reconstruction as compared with TMC and AVB.

Although there is no significant difference in biomechanical properties between TMC and AVB, inconsistent clinical complications have been reported. Mark et al. [1] reported that AVB had a higher subsidence and revision surgery rate compared with TMC. It was found in our study that there was a remarkable difference in stress distribution between these prostheses and the adjacent endplates. In TMC and AVB, the peak stress on the L2 caudal and L4 cranial endplates occurred at left or right rotation, which was much higher than that in HAVB model, while the HAVB model and the INTACT model showed the similar stress value and distribution in all motions. The stress on HAVB also demonstrated the similar trend that the maximum stress occurred at flexion and rotation, indicating that the implants and the interfaced endplate bear more stress in flexion or rotation than that in any other motions. Therefore, more attention should be paid to the protection of flexion and rotation during postoperative rehabilitation training.

A tremendous amount of force concentration was observed at one point of the spikes in AVB, and this may be useful in explaining the incidence of adjacent vertebral fracture and low back pain after surgery. While an astonishing force concentration was detected in TMC and AVB, the peak stress value on HAVB was much lower, probably due to two main reasons. One is that the material of n-HA/PA66 has a similar elasticity modulus with our human cortical bone, and this can effectively reduce the stress shielding effect. The other is the morphological design of HAVB that enlarges the contact surface with the endplate, which helps disperse the stress loaded on the prosthesis. Given some advantageous biomechanical properties over TMC and AVB, HAVB can be used as a viable option for spinal stability reconstruction.

The result of biomechanical analysis in this study demonstrates that this novel prosthesis of HAVB made of n-HA/PA66 has the similar stress value and distribution compared with INTACT model in all motions, indicating that the material of n-HA/PA66 is a viable material for bone tissue implantations. In addition, some other new bioceramics have been studied recently and enhanced with different nanocomposties [9, 21, 37]. For example, Khandan et al. [7, 8] studied the mechanical and biological properties of the bredigite-magnetite nanocomposite with various amounts of magnetite and found that the bredigite-30 wt% magnetite was an optimal sample with a fracture toughness of 2.69 MPa m1/2 and a Young’s modulus of 29 GPa. Its excellent biomechanical properties make it a suitable candidate for bone implantations. It is therefore warranted to pay more attention to these newly developed biocomposites.

There are several limitations in our study. First, as the two components of the HAVB system are rigidly fixed with no relative sliding in all loading conditions, it may not reflect the real clinical situation. In addition, we failed to consider the effect of bone grafting or bone cement filling in the prosthesis, knowing that they may also affect spinal stability. Finally, as the material properties applied to the element of the FE model do not exactly reflect the real behavior of the human lumbar spine, the result of FE analysis should be interpreted as a trend only, and further in vitro and vivo studies are required.

## Conclusion

The present study has demonstrated that HAVB has the similar biomechanical efficacy in spinal stability reconstruction as compared with TMC and AVB. The difference in clinical complications between TMC and AVB can be explained by the differential stress values and stress distributions on these prostheses. More attention should be paid to the protection of flexion and rotation during postoperative rehabilitation training. The material and configuration design of the prosthesis is closely related to the complications associated with the implant. Given some advantageous biomechanical properties over TMC and AVB, HAVB may prove to be a promising implant for spinal column reconstruction. Further in vivo and vitro studies are still required to validate our findings and conclusions.

## Additional files

**Additional file 1: Figure S1.** Comparison of ROM between the current FE model and Shim’s model in six different working conditions**.**

## Data Availability

The datasets used and/or analyzed during the current study are available from the corresponding author on reasonable request.

## Abbreviations

ALL: Anterior longitudinal ligament
AVB: Artificial vertebral body
CL: Capsular ligaments
FE: Finite element
HAVB: Height-adjustable vertebral body
ISL: Interspinous ligaments
ITL: Intertransverse ligaments
LF: Ligamentous flavum
n-HA/PA66: Nano-hydroxyapatite/polyamide66
PLL: Posterior longitudinal ligament
ROM: Range of motion
SSL: Supraspinous ligaments
TMC: Titanium mesh cage
