# Supplementary figures and images for: Biomechanical analysis of a novel height-adjustable nano-hydroxyapatite/polyamide-66 vertebral body: a finite element study
Source: J Orthop Surg Res. 2019 Nov 14;14:368. doi: 10.1186/s13018-019-1432-2 (PMC6854736; doi:10.1186/s13018-019-1432-2)

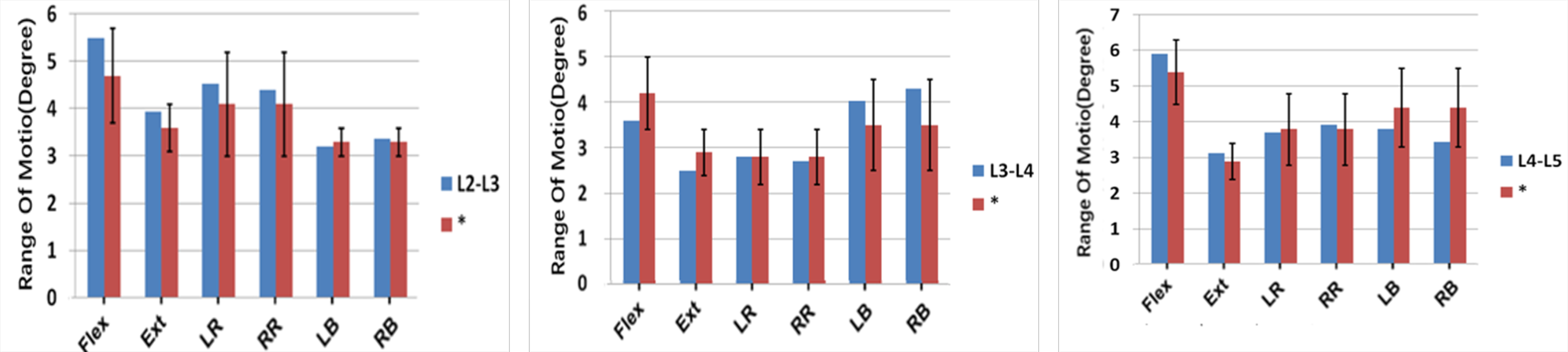

Supplement: Supplementary file 1 — Additional file 1: Figure S1. Comparison of ROM between the current FE model and Shim’s model in six different working conditions. [file 13018_2019_1432_MOESM1_ESM.tif]
